# Self-assembled BiFeO_3_@MIL-101 nanocomposite for antimicrobial applications under natural sunlight

**DOI:** 10.1186/s11671-023-03883-9

**Published:** 2023-09-11

**Authors:** Luca Pulvirenti, Cinzia Lombardo, Mario Salmeri, Corrado Bongiorno, Giovanni Mannino, Francesca Lo Presti, Maria Teresa Cambria, Guglielmo Guido Condorelli

**Affiliations:** 1 Dipartimento di Scienze Chimiche, Università degli Studi di Catania, Viale Andrea Doria 6, 95125 Catania, Italy; 2 Dipartimento di Scienze Biomediche e Biotecnologiche, Università degli Studi di Catania, Via S. Sofia 97, 95125 Catania, Italy; 3 CNR-IMM, Zona Industriale Strada VIII 5, 95121 Catania, Italy; 4 Consorzio INSTM UdR di Catania, Catania, Italy

**Keywords:** MOF, Ferrites, Hybrid material, Photo-activity, *E. coli*

## Abstract

In this paper, we report on the synthesis of a new hybrid photocatalytic material activated by natural sunlight irradiation. The material consists of multiferroic nanoparticles of bismuth ferrite (BFO) modified through the growth of the Fe-based MIL-101 framework. Material characterization, conducted using various techniques (X-ray diffraction, transmission electron microscopy, FTIR, and X-ray photoelectron spectroscopies), confirmed the growth of the MIL-101 metal–organic framework on the BFO surface. The obtained system possesses the intrinsic photo-degradative properties of BFO nanoparticles significantly enhanced by the presence of MIL-101. The photocatalytic activity of this material was tested in antibacterial experiments conducted under natural sunlight exposure within the nanocomposite concentration range of 100–0.20 µg/ml. The MIL-modified BFO showed a significant decrease in both Minimum Inhibiting Concentration and Minimum Bactericide Concentration values compared to bare nanoparticles. This confirms the photo-activating effect of the MIL-101 modification. In particular, they show an increased antimicrobial activity against the tested Gram-positive species and the ability to begin to inhibit the growth of the four *Escherichia coli* strains, although at the maximum concentration tested. These results suggest that the new nanocomposite BiFeO_3_@MOF has been successfully developed and has proven to be an effective antibacterial agent against a wide range of microorganisms and a potential candidate in disinfection processes.

## Introduction

Antibiotic resistance is a critical challenge for modern medicine [1, 2], which requires the development of efficient strategies and new advanced materials to control bacterial infections that are a serious threat to human health [3]. In this context, hybrid and inorganic nanomaterials have often been used for various therapeutic applications, ranging from imaging and diagnostics to cancer therapy and antimicrobial activity [4–9]. Among antimicrobial materials, photoactive compounds have been extensively studied [10, 11] for their tunable activity. The irradiation of optical-active compounds causes the production of large quantities of various reactive oxygen species (ROS). ROS exert antimicrobial activity, managing to attack a wide range of cellular targets, causing oxidative damage to DNA, lipids, proteins, and other cellular components [12, 13]. The result is extensive cellular damage leading to microbial cell death. Bacteria can counteract low levels of ROS via endogenous antioxidant defenses [14], including catalase enzymes, peroxidases, and superoxide dismutase, which aim to remove superoxide radicals and hydrogen peroxide. However, when an excessive production of ROS occurs, the intracellular redox state is modified, resulting in oxidative stress and an attack on lipids and various membrane constituents. The integrity of the bacterial membrane is thus altered, facilitating cell penetration of various reactive species, and, in turn, resulting in DNA damage and cell death [15]. However, these photoactive materials must be irradiated with a very intense visible light source or UV radiation to be truly efficient [16, 17]. This result is not always easy to achieve, and scientific research is focusing on the design and implementation of materials that can perform their action even under mild exposure conditions. Bismuth ferrite is a photoactive material [18, 19] for pollutant degradation and antibacterial activity under mild irradiation [20–23] because its small band gap (about 2.3 eV) makes the absorption of visible light possible. However, its activity is low because of the fast recombination of the photogenerated electron–hole pairs [18]. Photo-sensibilization through surface modification is one of the approaches studied to improve BFO activity [22–25]. Some studies have suggested that the combination of ferrite and MOF enhances catalytic efficiency and reactive oxygen species (ROS) production [26]. In particular, the functionalization of an iron-based MIL (MIL 53) with BFO nanoparticles has been found to reduce electron–hole recombination [27], thus improving the overall photoactivity.

In this article, we report a novel synthetic strategy to grow carboxylate-based MOF (MIL-101) on the surface of BFO nanoparticles. Iron-based MILs are a family of metal–organic frameworks formed by the assembly of trivalent clusters of Fe^3+^ and 2-aminoterephthalic acid as an organic ligand [28, 29], which exist in various isomeric forms. The most common isomers include MIL-88B, MIL-101, and MIL-53. Previous papers [30–33] investigated the possibility to grow metal–organic frameworks on metal oxides, but as far as we know no paper has reported on their growth on BFO particles. In addition, most of the adopted synthetic strategies caused the increase of nanoparticle dimensions obtaining sizes between 200 and 500 nm. This new MIL-101@BFO system aims to increase the photoactive properties of BFO without increasing the original size of the BFO nanoparticles. In order to achieve this result, we used a similar approach to that reported in our previous paper [4] for the growth of amorphous MOF-modified iron oxide nanoparticles. In this paper, BFO particles were used as the source of metal ions for the nucleation and growth of crystalline MIL-101. The effect of the growth process on particles size, morphology, and composition was investigated for different growth times (2 h and 4 h) through complementary surface and bulk characterization techniques: X-ray photoelectron spectroscopy (XPS), scanning electron microscopy (SEM), transmission electron microscopies (TEM), X-ray diffraction (XRD), and Fourier transmission infrared spectroscopy (FTIR). The improved photoactivity as an anti-bacterial material under natural sunlight radiation of the new BFO@MIL-101 nanosystem compared to bare BFO nanoparticles was proven using several bacterial strains of *Staphylococcus aureus*, *Staphylococcus haemolyticus,* and *Escherichia coli*.

## Materials and methods

### Materials

Bismuth nitrate [Bi(NO_3_)_3_·5H_2_O], iron nitrate [Fe(NO_3_)_3_·9H_2_O], iron (III) chloride (FeCl_3_·6H_2_O), L-tartaric acid [HO_2_CCH(OH)CH(OH)CO_2_H], 2-aminoterephthalic acid [(H_2_NC_6_H_3_-1,4-(CO_2_H)_2_], ethanol (CH_3_CH_2_OH), and dimethylformamide (DMF) were purchased from Sigma-Aldrich (Milan, Italy) and used as received. Water was of Milli-Q grade (18.2 MΩ cm) and was filtered through a 0.22-mm filter. For the evaluation of antibacterial activity, ATCC microorganisms were purchased from the American Type Culture Collection (Rockville, MD) and used as standard strains. Bacterial strains were *Escherichia coli* (ATCC 25922, ATCC 35218, ATCC 8739, ATCC 11229), *Staphylococcus aureus* (ATCC 29213, ATCC 29923), and *Staphylococcus haemolyticus* (ATCC 29970, ATCC 31174).

Culture mediums (CAMHB and MH agar), 0.9% w/v saline solution (OXOID), and 96-well polystyrene microtiter plates (Corning [TM]) were also purchased from Thermo Fisher Scientific.

### Synthesis of BiFeO_3_ nanoparticles (BFO)

BFO nanoparticles were synthesized by the simple sol–gel technique. In this process, 5.005 mmol of bismuth nitrate hexahydrate and 5 mmol of iron nitrate nonahydrate are solubilized in 50 mL of distilled water. Bismuth nitrate is only slightly soluble in water; therefore, some drops of nitric acid are added. Excess iron nitrate is weighed to compensate for its loss during the synthesis process due to its volatility. To this, 5 mmol of tartaric acid, used as a chelating agent, is added to the above solution and vigorously stirred for 1 h to obtain a homogeneous compound. Finally, the sol is placed in a hot plate and heated to 80 °C to obtain a gel and dried to a powder, which is subsequently annealed at 600 °C for 2 h to obtain the required BFO phase.

### Functionalization with iron-based MOF

The MIL structures with which bismuth ferrite nanoparticles are functionalized are obtained through a direct growth process. Two batches of nanoparticles are suspended in two beakers containing 15 ml of DMF each in which 1 mmol of 2-aminotherephthalic acid and 0.1 mmol of Iron (III) chloride hexahydrate had been previously solubilized. The reaction was kept in reflux for 4 h in an oil bath at 90 °C for one of the two beakers and for 2 h in the others. The functionalized nanoparticles are separated by centrifugation and rinsed several times in ethanol and water.

### Characterization

The structural, chemical, and morphological characterizations of the obtained materials were obtained through XRD, XPS, FTIR, SEM, and TEM analyses performed using methodologies described in our previous paper [6]. In particular, XRD was carried out with an XRD Smartlab Rigaku diffractometer equipped with a rotating anode of Cu Kα source radiation at 45 kV and 200 mA. XPS analysis was performed at a photoelectron take-off angle of 45° (relative to the sample surface) with a PHI 5000 Versa Probe Instrument using a monochromatic Al Kα X-ray source excited with a micro-focused electron beam. The XPS binding energy (B.E.) scale was calibrated on the C 1*s* peak of adventitious carbon at 285.0 eV. Transmission FTIR measurements of samples in KBr pellets were recorded using a JASCO FTIR 4600LE spectrometer in the spectral range 560–4000 cm^−1^ (resolution 4 cm^−1^). SEM and TEM images were obtained, respectively, using a field emission scanning electron microscope (FESEM) ZEISS VP 55 and a JEOL JEM 2010F working at 200 kV accelerating voltage. For TEM analysis, the powder was dispersed mechanically on an ultra-thin carbon-coated lacey carbon grid.

### Antibacterial activity

The Minimum Inhibitory Concentration (MIC) values of the BFO and BFO@MIL-101 nanocompounds were performed using the method of microdilutions in a liquid medium according to the standard procedures established by the Clinical and Laboratory Standards Institute (CLSI) [34].

Bacteria were grown overnight and diluted in CAMHB to obtain a cell density of 5 × 10^4^ CFU/ml in a 96-well microtiter plate. Then, 200 μL of the dispersed sample of BFO and BFO@MIL-101 was inoculated in the 96-well microtiter plate and subjected to serial doubling dilutions in the range of [100–0.20 µg/ml].

In order to evaluate the photocatalytic activity of the materials, three microplates containing the serial dilutions were incubated at 37 °C for 24 h in the dark; three microplates were, conversely, subjected to illumination with natural sunlight for 180 min. The average irradiance on the cell culture was measured using a PM160 T optical power meter from Thorlabs, and it was estimated to be in the 40–50 mW/cm^−2^ range and the ambient temperature was in the 25–27 °C range. Subsequently, the microplates were incubated at 37 °C for 24 h followed by optical density measurements at 600 nm using a microplate reader.

The Minimum Bactericidal Concentration (MBC), which represents the lowest concentration of antimicrobial agent capable of killing 99.9% of bacteria, was determined. Then 100 µl was taken from the wells in which no visual growth was observed and subcultures were prepared on MH agar plates. The plates, incubated at 37 °C for 24 h, were observed to evaluate the possible presence of bacterial growth. Gram-negative *E. coli* and Gram-positive *Staphylococci* were used as model bacteria.

## Results and discussion

**Fig. 1:**
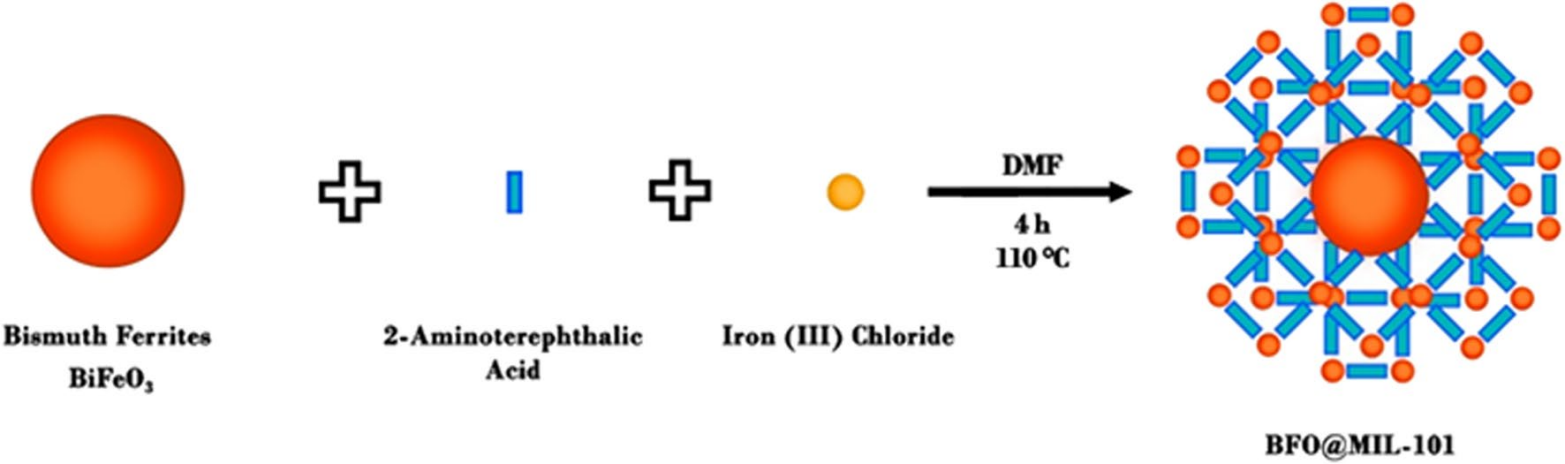
Schematic representation for the synthesis of hybrid nanomaterial BFO@MIL-101

The adopted method for the synthesis of MOF-functionalized bismuth ferrite nanoparticles (BFO) is schematized in Fig. 1. The adopted pathway has as a key point the use of BFO inorganic nanoparticles as both Fe^3+^ ion source and nucleation center for the growth of the MIL structure. The first step of this route is the synthesis of BFO particles by a sol–gel method, followed by surface modification through the growth of a metal–organic framework using 2-aminoterephthalic acid as organic precursor. A small amount of FeCl_3_ (FeCl_3_/aminoterephthalic acid atomic ratio 1/10) was also added to the reaction mixture to favor MIL-101 growth [4]. Details of the synthetic procedure are described in the materials and methods section.

**Fig. 2:**
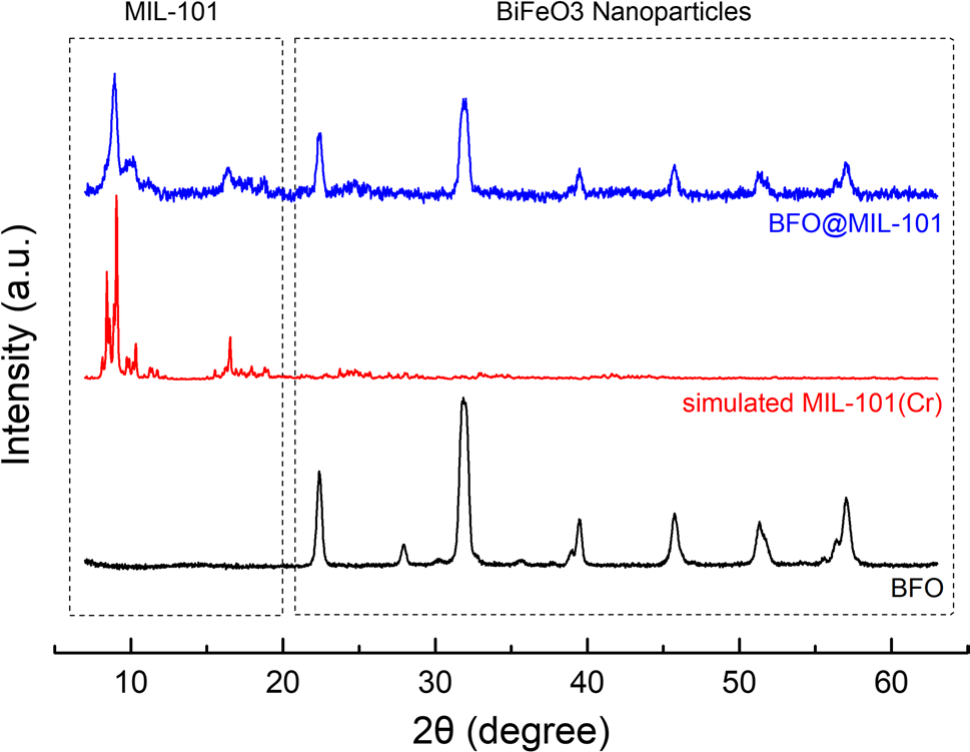
X-ray patterns of bare bismuth ferrite powder (black line), simulated MIL-101(Cr) (red line) and BFO@MIL-101 (blue line)

The XRD patterns of BFO before and after the MOF growth process (growth time 4 h) are shown in Fig. 2. The main diffraction peaks at low angles (8.9°, 9.8°, and 16.4°) are typical of the MIL-101 family [35, 36], and the novel material exhibits a very similar profile to the MIL-101 simulated pattern. The peaks above 20° are instead assignable to the BFO phase [37]. The presence of the diffraction peaks of both precursors is a clear indication that the growth process has occurred successfully and without degradation of the crystalline structure of the inorganic core.

**Fig. 3:**
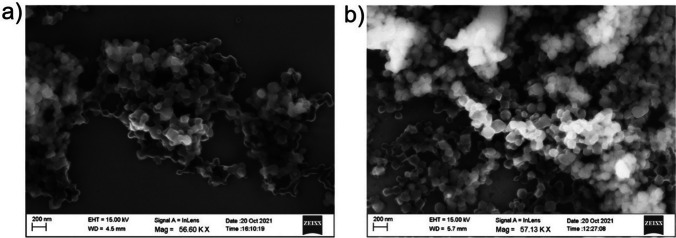
SEM morphology of **a** bare bismuth ferrite and **b** BFO@MIL-101

The morphology and size of the nanoparticles of BFO@MIL-101 and bare BFO, used as a reference sample, were observed by electron scanning microscopy (SEM). In both cases, grains of average size around 100–200 nm were visible in the images. There were no evident morphological differences between the two samples, although the grains of the BFO@MIL-101 appeared to be squared (Fig. 3).

The atomic composition of BFO surfaces before and after MIL growth was investigated by XPS. The values obtained from the quantitative analysis are given in Table 1. After the formation of the MIL coating, the C concentration was increased, as expected for the formation of an organic layer.

**Table 1:** XPS atomic concentration of BFO and BFO@MIL-101 particles

	XPS atomic concentrations						
	C 1*s*	O 1*s*	Fe 2*p*	Bi 4*f*	N 1*s*	Cl 2*p*	Bi/Fe
BFO	30.30	51.10	6.60	12.1	–	–	1.80
BFO@MIL-101	52.76	37.92	4.40	0.10	3.71	1.11	0.002

**Fig. 4:**
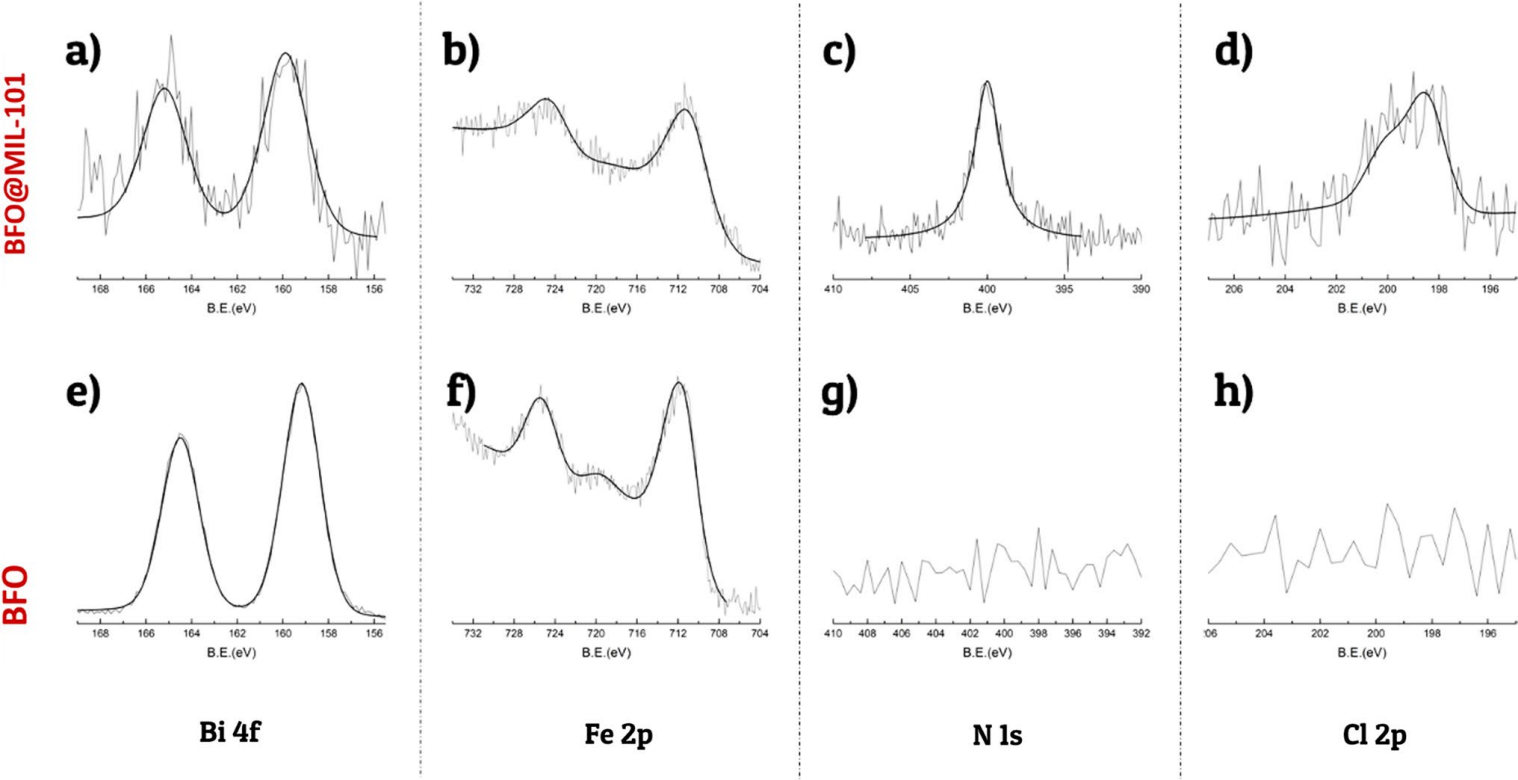
XPS spectra of Bi 4*f* (**a**, **e**), Fe 2*p* (**b**, **f**), N 1*s* (**c**, **g**) and Cl 2*p* (**d**, **h**), of MNPs@MIL (**a**–**d**) and bare MNPs (**e**–**h**)

**Fig. 5:**
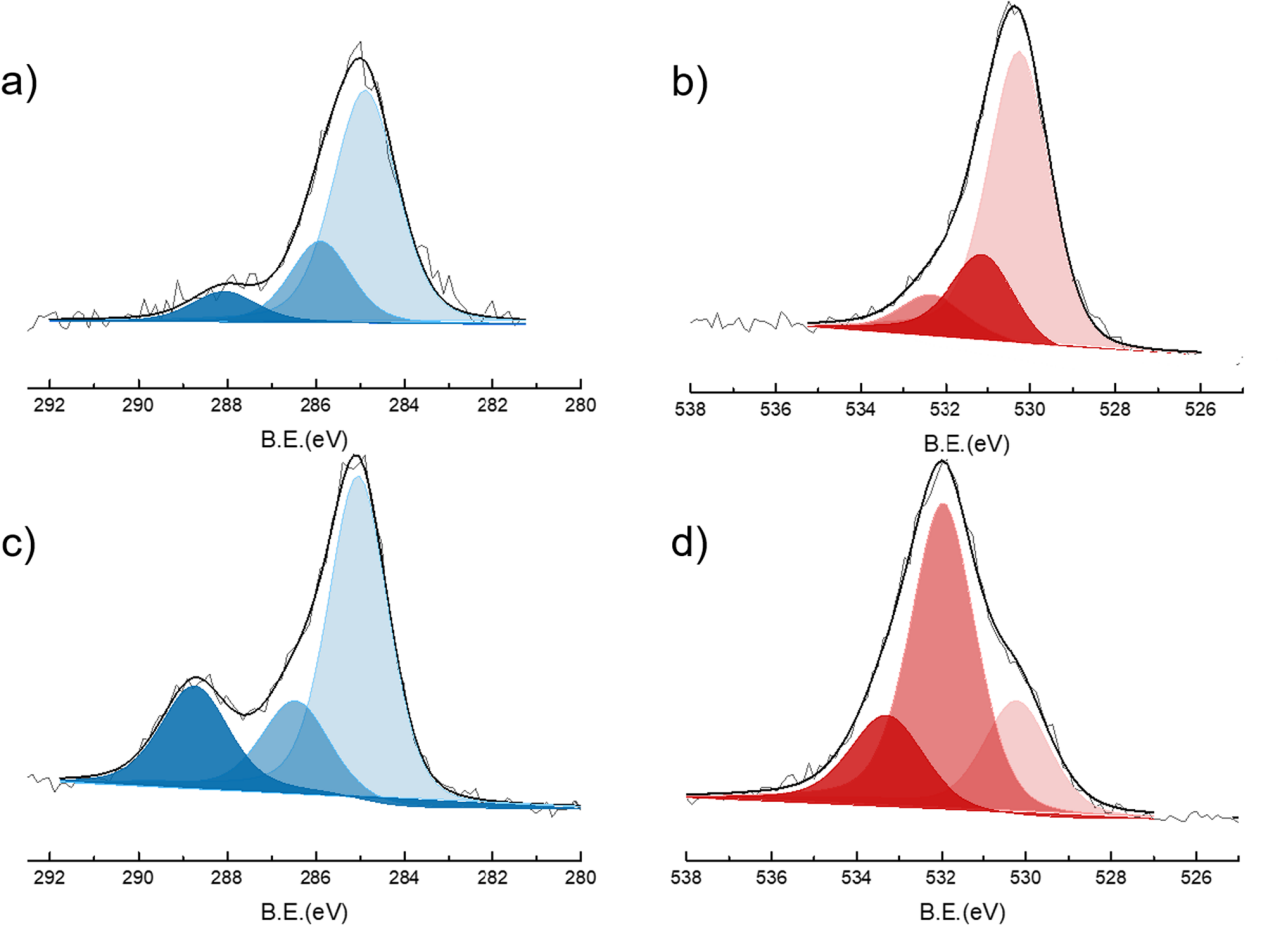
XPS spectra of C 1*s* (**a**, **c**) and O 1*s* (**b**, **d**) of bare MNPs (**a**, **b**) and MNPs@MIL-101 (**c**, **d**)

Before MIL growth the surface Bi/Fe atomic ratio was about 1.8, slightly higher than the stoichiometric bulk ratio due to Bi surface segregation often observed for BFO nanoparticles [25, 38]. After MIL growth both iron and bismuth concentrations decreased. The iron concentration decreased slightly because of the lower iron density in the MIL structure, while the bismuth concentration decreased significantly (Bi/Fe atomic ratio about 0.002), thus indicating the formation of an iron-based MIL coating on the BFO surface. A significant amount of nitrogen appeared in the BFO@MIL-101 spectrum, due to the amino groups of aminoterephthalate ligands [38]. Another difference between the modified sample and BFO bare is the presence of chlorine, which is in the coordination sphere of the iron cluster, according to the MIL structure [39]. High-resolution spectra of Bi 4*f*, Fe 2*p*3/2, N 1*s*, Cl 2*p*, C 1*s*, and O 1*s* regions before and after MOF modification are shown in Figs. 4 and 5. The positions of Bi 4*f* 7/2 and 5/2 peaks of bare BFO are 164.5 eV and 154.2 eV, respectively, consistent with the presence of Bi^3+^ [40].

After MOF modification the peak position does not change, but the intensity is strongly decreased due to the MOF formation as mentioned above. In the Fe2*p* spectrum for bare BFO (Fig. 4f) two peaks appeared at 711.9 and 725.7 eV, corresponding to Fe 2*p*_3/2_ and Fe 2*p*_1/2_ signals [41]. After MIL growth the centroid of the peak at 711.9 eV is shifted (Fig. 4b), likely due to the convolution of Fe^3+^ in the MIL structure (typically around 712.6 eV) and the Fe^3+^ ions of BFO. The evolutions of the C 1*s* and O 1*s* signals before and after the formation of the MIL coating are shown in Fig. 5. Before growing MIL, the C 1*s* signal of bare BFO is due to the adventitious carbon that is always present in XPS spectra. It consists of a main peak at 285 eV and a small component at 286.0 eV due to oxidized carbons [42, 43]. There is also a low-intensity tail around 288 eV, probably due to surface carbonate formation. The O 1*s* signal of bare BFO is dominated by a component at 530.2 eV due to the oxygen of the ferrite. Higher B.E. components at 531.1 eV and 532.4 eV are due to surface hydroxylic groups. After MIL formation, the shape of the C 1*s* peak is changed and three characteristic components at 285, 286.5, and 288.8 eV can be observed. The highest peak at 285 eV is assigned to aliphatic and aromatic hydrocarbon atoms due to the aromatic ring of the terephthalic acid and to adventitious carbon. The component centered at 286.5 eV is due to both the C-O groups and the C-N atoms of the amino-terephthalic moieties. The characteristic peak at 288.6 eV is due to carboxylate groups (-COO) of the terephthalate ligand [40]. Analogously, the O 1*s* spectral region of MIL-101@BFO composite shows a different shape compared to the one of bare BFO. In particular, the component at 530.2 eV assigned to the oxygen of the ferrite is strongly decreased consistently with the growth of Fe-based MIL-101 at BFO expense. The most intense component at 532.0 eV is due to the oxygen atoms of the carboxylate groups of the terephthalate ligand, and the third component at 533.3 eV can be assigned to adsorbed water.

**Fig. 6:**
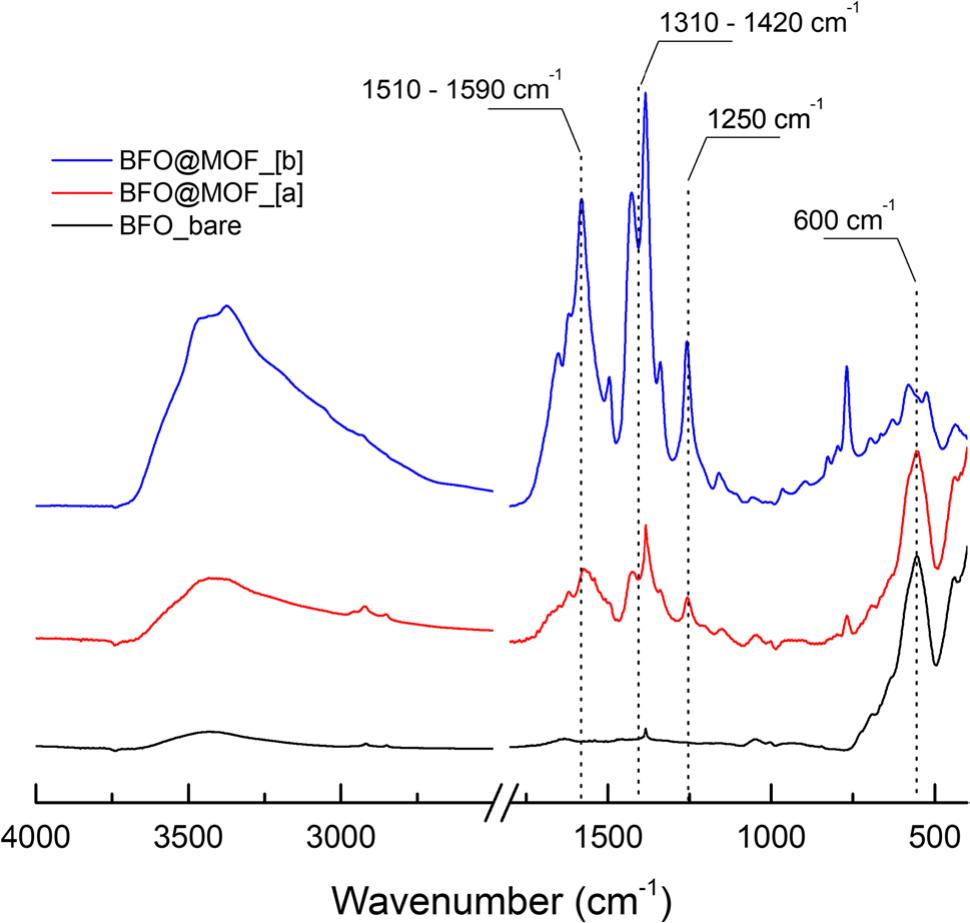
FTIR spectra of bismuth ferrite nanoparticles (black line), hybrid BFO@MIL-101 after 2 h (red line) and hybrid BFO@MIL-101 after 4 h (blue line)

In order to understand the effect of time on the MOF growth process, syntheses with various duration were performed. In particular, Fig. 6 reports the comparison between FTIR spectra of the samples obtained with reaction times of 2 h (BFO@MIL[a]) and 4 h (BFO@MIL[b]). The spectrum of bare BFO nanoparticles is added for comparison. The intense peak at 600 cm^−1^ is common to all three samples and is related to Fe − O stretching modes. This peak, typical for iron oxides, appears to be less intense in the BFO@MIL[b] sample due to the increased organic coverage. The strong bands at 1250 cm^−1^, 1310–1420 cm^−1^, and 1510–1590 cm^−1^ are the typical vibrational modes of the aminoterephthalate ligands and can be assigned, respectively, to the C–N stretching of the amino group and to the COO^−^ symmetric and asymmetric stretching of the terephthalate [36, 44]. It can be noted that peaks in this area are more intense and sharp in the BFO@MIL[b] sample suggesting the presence of a thicker MOF coating compared to BFO@MIL[a]. Finally, the peaks at 3456 cm^−1^ and 3373 cm^−1^, in both modified samples, can be assigned to the asymmetrical and symmetrical stretching of the amine groups. These peaks overlap the broad band in the 3000–3500 region due to the O–H stretching of water.

**Fig. 7:**
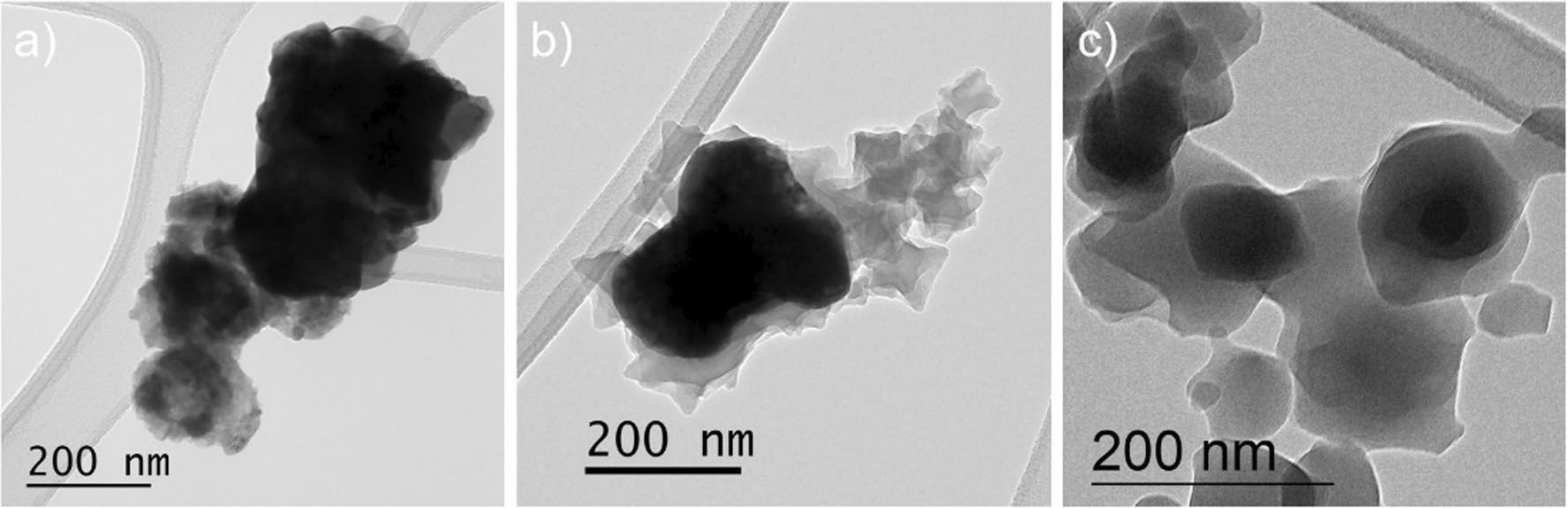
TEM images of **a** bare bismuth ferrite, **b** hybrid BFO@MIL-101 after 2 h and **c** hybrid BFO@MIL-101 after 4 h

The dependence of MOF growth on the reaction time is also shown by a morphological study (Fig. 7a–c) performed using transmission electron microscopy (TEM). The bare BFO particles (Fig. 7a) are covered with small MOF crystals already after 2 h of reaction (Fig. 7b).

After 4 h of reaction (Fig. 7c), the BFO@MIL[b] sample has a thicker and more homogeneous coating than the BFO@MIL[a]. TEM images show less dense structures due to the presence of MOF and small darker features due to residual BFO inside the MOF. Note that after 4 h nanoparticle dimensions are smaller than the ones of the pristine BFO particles.

Antimicrobial tests were performed to evaluate photoactive properties. The antibacterial activity of BFO and BFO@MIL-101 was tested in duplicate on four strains of Staphylococci (species Gram-positive) and four strains of *E. coli* (species Gram-negative). Table 2 shows the values of Minimum Inhibitory Concentration (MIC) and Minimum Bactericidal Concentration (MBC) obtained after 24 h of bacterial incubation with the two nanoparticles in the dark and following exposure to natural sunlight. As shown in the table, both nanoparticles did not exhibit any bacteriostatic or bactericidal activity in the dark condition, even at the highest concentration tested (100 µg/ml). The antibacterial activity of both compounds showed a significant increase (in terms of decrease of MIC and MBC values) following irradiation with natural sunlight, which confirms the photoactivity of these materials.

**Table 2:** Minimum Inhibitory Concentration (MIC) and Minimum Bactericidal Concentration (MBC) of BFO and BFO@MIL in dark conditions and under sunlight irradiation

Bacterial strains	Dark				Sunlight			
	BFO		BFO@MIL-101		BFO		BFO@MIL-101	
	MIC (µg/mL)	MBC (µg/mL)	MIC (µg/mL)	MBC (µg/mL)	MIC (µg/mL)	MBC (µg/mL)	MIC (µg/mL)	MBC (µg/mL)
*S. aureus* ATCC 29213	> 100	> 100	> 100	> 100	0.20	> 100	0.20	0.20
*S. aureus* ATCC 29923	> 100	> 100	> 100	> 100	0.39	> 100	0.20	0.20
*S. haemolyticus* ATCC 29970	> 100	> 100	> 100	> 100	0.39	> 100	0.20	0.39
*S. haemolyticus* ATCC 31874	> 100	> 100	> 100	> 100	0.39	> 100	0.20	0.39
*E. coli* ATCC 25922	> 100	> 100	> 100	> 100	> 100	> 100	100	> 100
*E. coli* ATCC 35218	> 100	> 100	> 100	> 100	> 100	> 100	100	> 100
*E. coli* ATCC 8739	> 100	> 100	> 100	> 100	> 100	> 100	100	> 100
*E. coli* ATCC 11229	> 100	> 100	> 100	> 100	> 100	> 100	100	> 100

The data obtained in this study show that BFO@MIL-101 has a better antibacterial profile when compared to unmodified BFO, as it exhibits increased antibacterial activity against both the Gram-positive and Gram-negative species tested. In particular, bare BFO has a reduction in the MIC value (from > 100 µg/ml in dark condition to 0.20 or 0.39 µg/ml under irradiation) for all strains of *Staphylococcus* tested. This value is significantly lower for BFO@MIL-101 (from > 100 µg/ml in dark conditions to 0.20 µg/ml under irradiation). Therefore, compared to other previously reported active materials, it shows an enhancement of the antimicrobial activity against the bacterial strains of *Staphylococcus aureus* (coagulase-positive), *Staphylococcus haemolyticus* (coagulase-negative), and *Escherichia coli* [9, 45].

In addition, under irradiation BFO@MIL-101 nanoparticles have lower MBC values (0.20 or 0.39 µg/ml) compared to bare BFO (> 100 µg/ml) on all strains of *Staphylococcus* tested. However, the most relevant aspect of BFO@MIL is the ability to begin inhibiting the growth of the four *E.coli* strains, albeit at the highest concentration tested (100 µg/ml). Despite the presence of lipopolysaccharide in the outer membrane of Gram-negative bacteria, which gives protection against various antimicrobial agents, BFO@MIL-101 nanoparticles photoactivated with natural sunlight, unlike nanoparticles, equally photoactivated, of pure BFO, are able to exert a bacteriostatic action (inhibiting bacterial growth) on all four strains of *E.coli* used in the study. A sample is considered bactericidal when the ratio MBC/MIC (tolerance factor) is ≤ 4 and bacteriostatic when this ratio is > 4. Table 2 provides evidence of a bactericidal effect of BFO@MIL-101 in the tested strains of *Staphylococcus aureus* and *Staphylococcus haemolyticus*, tolerance factor of 1 and 1.95, respectively [46]. However, it was not possible to calculate the tolerance factor for the E. coli strains since the MBC value was not defined.

The improvement obtained after MOF functionalization suggests that the antibacterial properties of BFO resulting from photoactivation with natural sunlight are substantially improved by MOF functionalization.

## Conclusions

This study reports the synthesis of a new hybrid material obtained through the direct growth of crystalline MIL-101 on bismuth ferrite nanoparticles. FTIR spectroscopy and transmission electron microscopy (TEM) have shown that the growth of MOF on the composite material is dependent on the reaction time. In addition, the morphological analysis conducted with TEM suggested that the growth of MIL-101 occurred at the expense of the bismuth ferrite inorganic core. BFO@MIL-101 was applied as an anti-bacterial material under natural sunlight radiation against the bacterial strains of *S. aureus*, *S. haemolyticus,* and *E. coli*. Tests have shown that the hybrid nanomaterial possesses a significantly increased antibacterial photoactivity, in terms of reduction of MIC and MBC values, compared to bare BFO. In particular, BFO@MIL-101 showed a lower MIC (0.20 µg/ml) and MBC (0.20 or 0.39 µg/ml) compared with bare BFO (0.20 or 0.39 µg/ml and > 100 µg/ml, respectively) for selected strains of *Staphylococcus* tested. Furthermore, another important aspect of BFO@MIL-101 is the capability to inhibit four *E.coli* strains at the highest concentration tested (100 µg/ml). The data obtained on antibacterial activity are promising, making the material suitable for use in disinfection processes against a wide range of bacterial strains to prevent and control the persistence of bacterial contaminations.

## Data Availability

The data presented in this study are available from the corresponding author upon reasonable request.
